# Supplementary material for: Strengthening the community governance of healthcare services in ‘fragile’ settings: Evidence from Burundi and South Kivu, DR Congo
Source: PLOS Glob Public Health. 2023 Aug 15;3(8):e0001697. doi: 10.1371/journal.pgph.0001697 (PMC10427014; doi:10.1371/journal.pgph.0001697)
Supplement: S1 Table — (DOCX) [file pgph.0001697.s001.docx]

**S1 Table**. Indicators for impact evaluation

|  | *Index* | *Indicators* | *Source* |
| --- | --- | --- | --- |
| Community Governance | 1.  HFC organisation | (1) HFC meetings per month; (2) consensus-based decision-making process (binary); (3) terms of reference available (at time of visit); (4) HFC self-declared confidence in their work (5-item Likert scale); (5) HFC self-declared voice at HFC (5-item Likert scale); (6) existence of executive committee; coding of the question ‘What are the main activities of the HFC?’ - binary variables divided between (7) 1 = broad general management and (8) 1 = precise management activities. | HFC/HF surveys HFC minutes |
|  | 2. Accountability | *2.a.* mean of mean made of  *HFC decision rights*: (1-6) HFC, HFC executives and HFC president decision rights in 7 areas, according to the chief nurse and HFC executives; as well as (7) HFC co-signs receipts (self-declared).  *Information sent by HFC*: (1) HFC information (minutes) sent to chief nurse and (2) health district, (3) months since last HFC general assembly. | HFC/HF surveys |
|  |  | *2.b.* mean score made of  *Information on user side (with HFC)*: (1) respondent knows HFC members; (2) went to HFC meeting last year, (3) asked the HFC a question last year, (4) identifies HFC as co-manager of the HF  *Information on user side (at HF)*: (1) understood the bill when visiting HF (5-item Likert scale); (2) staff gave explanations (5-item Likert scale); (3) prices were displayed during the visit (binary). | household surveys |
| Management | 3.  Management | *3.1. Drugs and equipment* (number of days of stock-out per month): mean of (1) drugs; (2) medical items (non-drugs); and (3) equipment. | HIS data |
|  |  | *3.2. Human resources*: (1) qualified staff; (2) support staff; (3) change in chief nurse in the last year (binary). | HFC/HF surveys |
|  |  | *3.3. Infrastructure*: (1) small building (e.g. shed, toilet, shower); (2) building (new); (3) provision of electricity and (4) water. | HFC/HF surveys |
|  |  | *3.4. Budget and organisation*: (1) part of user fees in revenue; (2) expenses/revenue ratio; (3) ratio of basic services available. | HFC/HF surveys |
| Service delivery | 4.  Quality | Experience at HF in last visit last year: (1) rating of quality (5-item Likert scale); (2) attention of staff (5-item Likert scale); (3) price paid (5-item Likert scale); and (4) use of drugs (5-item Likert scale). | household surveys |
|  | 5.  Equity in access | Household-level binary variables based on experience in the past year: (1) household faced catastrophic expenditure in health; (2) could not afford drugs; (3) could not afford care; (4) did go to the closest HF | household surveys |
|  | 6.  Provision of services | HF-declared number of (1) Institutional delivery; (2) curative visit, prenatal visit; (3) postnatal visit; (4) family planning (new users); (5) referral; (6) immunization of children (ATV 2-5). All relative to population of the catchment area.^1^ | HIS/PBF data |

Note: part of this table appears in a different paper discussing another aspect of the interventions (see endnote i). As provided in the health information systems. It is important to note that there are serious limitation to the use of this indicator as the denominator (the population) is notoriously imprecise
