# Supplementary material for: Strengthening the community governance of healthcare services in ‘fragile’ settings: Evidence from Burundi and South Kivu, DR Congo
Source: PLOS Glob Public Health. 2023 Aug 15;3(8):e0001697. doi: 10.1371/journal.pgph.0001697 (PMC10427014; doi:10.1371/journal.pgph.0001697)
Supplement: S2 Table — (DOCX) [file pgph.0001697.s002.docx]

**S2 Table** Situation at baseline

|  | control group | intervention^1^ | n |
| --- | --- | --- | --- |
| *basic controls* |  |  |  |
| Hills/villages represented in the HFC | 6.500  (0.377) | 6.198  (0.292) | 329 |
| Ratio of HFC members who are women | 0.361  (0.016) | 0.365  (0.013) | 329 |
| “ ” went to secondary school | 0.386  (0.022) | 0.358  (0.015) | 329 |
| Number of support staff at HF | 5.984  (0.259) | 5.623  (0.174) | 329 |
| Population in HF catchment area | 13,390  (739) | 14,127  (569) | 329 |
| Faith-based HF (*centre de santé agréé*) | 0.246  (0.039) | 0.237  (0.030) | 329 |
| HF located in urban or peri-urban area | 0.131  (0.031) | 0.072  (0.018) | 329 |
| *outcome indexes^2^* |  |  |  |
| 1. HFC organisation | -0.013  (0.030) | -0.028  (0.036) | 329 |
| 2.1. Accountability: HF indicators | 0.000  (0.040) | 0.052  (0.054) | 329 |
| 2.2. Accountability: household indicators | -0.007  (0.023) | -0.021  (0.029) | 4605 |
| 2.2.a HFC-population interactions | -0.088  (0.031) | -0.007  (0.038) | 3687 |
| 2.2.b Population information at HF | -0.020  (0.033) | -0.051  (0.044) | 3538 |
| 3.1. HF Management (mean of 4 indexes below) | -0.000  (0.034) | 0.014  0.044) | 329 |
| 3.1. HF management: equipment | 0.000  (0.061) | 0.067  (0.081) | 329 |
| 3.2. HF management: human resources | -0.000  (0.055) | -0.018  (0.071) | 329 |
| 3.3. HF management: infrastructure | -0.000  (0.074) | 0.078  (0.093) | 329 |
| 3.4. HF management: finance | 0.000  (0.043) | -0.072  (0.054) | 329 |
| 4. HF perceived quality | -0.023  (0.040) | 0.046  (0.052) | 3208 |
| 5. HF perceived access | -0.054  (0.032) | -0.067  (0.047) | 5991 |
| 6. Provision of services | -0.000  (0.064) | 0.076  (0.081) | 329 |

Standard errors in parentheses | 1. Calculated as c + β_1_ in the OLS/LPM: Y= c+βI+ +ε. reported significance level of β_1_: *<0.1, *<0.05, ***<0.001. None of the comparisons had a p-value < 0.1 | 2. See tables 1 and A1 for detail. | source: our own surveys at baseline as explained in the methods section of the article.
