# Supplementary material for: Strengthening the community governance of healthcare services in ‘fragile’ settings: Evidence from Burundi and South Kivu, DR Congo
Source: PLOS Glob Public Health. 2023 Aug 15;3(8):e0001697. doi: 10.1371/journal.pgph.0001697 (PMC10427014; doi:10.1371/journal.pgph.0001697)
Supplement: S3 Table — (DOCX) [file pgph.0001697.s003.docx]

**S3 Table**. Intent-to-Treat – main effects

|  | (1) | (2.1) | (2.2) | (3) | (4) | (5) | (6) |
| --- | --- | --- | --- | --- | --- | --- | --- |
|  | HFC  orga. | HFC  rights | HFC  reach | HF  mgmt | perceived  quality | access | service  delivery |
| **Diff-in-diff without controls** | | | | | | | |
| Intervention^a^ (ITT) | 0.167  (0.055) | 0.207  (0.086) | -0.030 (0.047) | 0.076 (0.055) | -0.045 (0.064) | 0.032 (0.050) | -0.024 (0.111) |
| [FWER p-val.]^x^ | [0.013] | [0.049] | [0.675] | [0.289] | [0.663] | [0.675] | [0.844] |
| Controls | No | no | no | no | no | no | no |
| district FE | No | no | no | no | no | no | no |
| N | 658 | 658 | 13043 | 658 | 10098 | 15667 | 658 |
| adj. R-sq. | 0.052 | 0.070 | ± | 0.019 | ± | ± | 0.000 |
| **Diff-in-diff with controls** | | | | | | | |
| Treat*time^a^ | 0.168  (0.053) | 0.210 (0.081) | -0.034 (0.048) | 0.076 (0.046) | -0.007 (0.064) | 0.021 (0.046) | -0.024 (0.080) |
| [FWER p-value]^x^ | [0.011] | [0.059] | [0.929] | [0.406] | [0.956] | [0.956] | [0.956] |
| controls | yes | yes | yes | yes | yes | yes | yes |
| district FE | yes | yes | yes | yes | yes | yes | yes |
| N | 656 | 656 | 12790 | 656 | 9864 | 15395 | 656 |
| adj. R-sq | 0.105 | 0.199 | ± | 0.289 | ± | ± | 0.508 |
| **ANCOVA** | | | | | | | |
| Treat^a^ | 0.126 (0.047) | 0.368 (0.080) |  | 0.086 (0.031) |  |  | 0.029 (0.055) |
| [FWER p-value] | [0.012] | [0] |  | [0.059] |  |  | [0.594] |
| z-score baseline | 0.183 (0.017) | 0.150  (0) |  | 0.352 (0.013) |  |  | 0.725 (0.596) |
| controls | no | no |  | no |  |  | no |
| district FE | no | no |  | no |  |  | no |
| N | 329 | 329 |  | 329 |  |  | 329 |
| adj. R-sq | 0.055 | 0.075 |  | 0.217 |  |  | 0.579 |

Note: Standard errors in parentheses. | see Tables 2 and A1 for the indicators that make each index, and Table A2 for robustness checks | a. β_1_ in model 1 | x. Family-Wise Error Rate p-value, bootstrapped 1001 times | ±. estimation for weighted and stratified sample, no adjusted R^2^ .
