# Supplementary material for: Strengthening the community governance of healthcare services in ‘fragile’ settings: Evidence from Burundi and South Kivu, DR Congo
Source: PLOS Glob Public Health. 2023 Aug 15;3(8):e0001697. doi: 10.1371/journal.pgph.0001697 (PMC10427014; doi:10.1371/journal.pgph.0001697)
Supplement: S4 Table — (DOCX) [file pgph.0001697.s004.docx]

**S4 Table**. Intent-to-Treat – main effects, by country

|  | (1) | (2.1) | (2.2) | (3) | (4) | (5) | (6) |
| --- | --- | --- | --- | --- | --- | --- | --- |
|  | HFC  orga. | HFC  rights | HFC  reach | HF  mgmt | perceived  quality | access | service  delivery |
| **Diff-in-diff without controls** | | | | | | | |
| effect in Burundi^a^ | 0.119  (0.056) | 0.083 (0.094) | -0.007 (0.050) | 0.061 (0.053) | -0.174 (0.076) | -0.030 (0.045) | -0.001 (0.141) |
| [FWER p-value]^x^ | [0.098] | [0.604] | [0.903] | [0.614] | [0.109] | [0.660] | [0.992] |
| extra effect Kivu^b^ | 0.222 (0.140) | 0.741  (0.210) | -0.094 (0.106) | 0.215 (0.137) | 0.223 (0.132) | 0.086 (0.107) | 0.055 (0.192) |
| [FWER p-value]^x^ | [0.312] | [0.002] | [0.368] | [0.368] | [0.240] | [0.431] | [0.773] |
| total effect Kivu^c^ | 0.342 (0.129) | 0.823 (0.187) | -0.102 (0.094) | 0.276 (0.127) | 0.049 (0.107) | 0.056 (0.097) | 0.054 (0.130) |
| [FWER p-value]^x^ | [0.027] | [0.001] | [0.327] | [0.078] | [0.791] | [0.733] | [0.791] |
| Kivu baseline diff.^d^ | -0.094 (0.075) | -0.001 (0.090) | 0.108  (0.042) | -0.400 (0.062) | -0.050 (0.078) | 0.543 (0.049) | -0.446 (0.099) |
| controls | no | no | no | no | no | no | no |
| district FE | no | no | no | no | no | no | no |
| N | 658 | 658 | 13,043 | 658 | 10,098 | 15,667 | 658 |
| adj. R-sq | 0.063 | 0.142 | ± | 0.193 | ± | ± | 0.057 |
| **Diff-in-diff with controls** | | | | | | | |
| effect in Burundi^a^ | 0.121 (0.054) | 0.088 (0.087) | 0.013 (0.050) | 0.061 (0.047) | -0.161 (0.075) | -0.030 (0.044) | -0.000 (0.097) |
| [FWER p-value]^x^ | [0.079] | [0.614] | [0.914] | [0.614] | [0.085] | [1] | [1] |
| extra effect Kivu^b^ | 0.221 (0.137) | 0.736 (0.204) | -0.125 (0.107) | 0.214 (0.123) | 0.227 (0.131) | 0.093 (0.110) | 0.054 (0.176) |
| [FWER p-value]^x^ | [0.298] | [0.004] | [0.417] | [0.291] | [0.249] | [0.455] | [0.774] |
| total effect Kivu^c^ | 0.342 (0.126) | 0.823 (0.185) | -0.111 (0.095) | 0.276 (0.147) | 0.066 (0.108) | 0.063 (0.100) | 0.054 (0.146) |
| [FWER p-value]^x^ | [0.016] | [0] | [0.297] | [0.037] | [0.666] | [0.666] | [0.992] |
| Kivu baseline diff.^d^ | -0.066 (0.120) | 0.183 (0.160) | 0.083 (0.089) | -0.451 (0.108) | -0.273 (0.099) | 0.512 (0.084) | -0.316 (0.183) |
| controls | yes | yes | yes | yes | yes | yes | yes |
| district FE | yes | yes | yes | yes | yes | yes | yes |
| N | 656 | 656 | 12790 | 656 | 9864 | 15395 | 656 |
| adj. R-sq | 0.112 | 0.264 | ± | 0.353 | ± | ± | 0.514 |
| **ANCOVA** | | | | | | | |
| effect in Burundi^a^ | 0.058 (0.050) | 0.166 (0.089) |  | 0.057 (0.034) |  |  | 0.029 (0.067) |
| [FWER p-value]^x^ | [0.472] | [0.232] |  | [0.472] |  |  | [0.666] |
| extra effect Kivu^b^ | 0.213 (0.108) | 0.633 (0.156) |  | 0.096 (0.071) |  |  | 0.000 (0.120) |
| [FWER p-value]^x^ | [0.143] | [0.001] |  | [0.554] |  |  | [0.997] |
| total effect Kivu^c^ | 0.272 (0.097) | 0.799 (0.128) |  | 0.153 (0.063) |  |  | 0.029 (0.098) |
| [FWER p-value]^x^ | [0.023] | [0] |  | [0.124] |  |  | [0.768] |
| Kivu baseline diff.^d^ | 0.183 (0.070) | 0.185 (0.068) |  | 0.419 (0.046) |  |  | 0.750 (0.059) |
| controls | no | no |  | no |  |  | no |
| district FE | no | no |  | no |  |  | no |
| N | 329 | 329 |  | 329 |  |  | 329 |
| adj. R-sq | 0.064 | 0.187 |  | 0.233 |  |  | 0.583 |

Note: Standard errors in parentheses | see Tables 2 and A1 for the indicators that make each index, and Table A2 for robustness checks | a, b, c, and d, are respectively, β_1_, β_2_, β_1_ + β_2_, and β_0_ in model 4 | x. Family-Wise Error Rate p-value, bootstrapped 1001 times | ±. estimation for weighted and stratified sample, no adjusted R^2^.
