# Supplementary material for: Strengthening the community governance of healthcare services in ‘fragile’ settings: Evidence from Burundi and South Kivu, DR Congo
Source: PLOS Glob Public Health. 2023 Aug 15;3(8):e0001697. doi: 10.1371/journal.pgph.0001697 (PMC10427014; doi:10.1371/journal.pgph.0001697)
Supplement: S5 Table — (DOCX) [file pgph.0001697.s005.docx]

**S5 Table**: Intent-to-Treat – effects for the social accountability indicators at HF-level (HF rights)

|  | (1) | (2) | (3) | (4) | (5) | (6) | (7) | (8) | (9) | (10) |
| --- | --- | --- | --- | --- | --- | --- | --- | --- | --- | --- |
| **diff-in-diff without controls** | | | | | | | | | | |
| decision rights *of*  according to (see table1) | HFC pres.  HFC | HFC pres.  chief nurse | HFC exec.  HFC | HFC exec.  chief nurse | HFC  HFC | HFC  chief nurse | HFC co-signs  HF orders | HFC minutes  shared w. HF | HFC minutes  shared w. HF | HFC met pop. |
| effect in Burundi^a^ | -0.005 (0.074) | 0.051  (0.069) | 0.015  (0.070) | 0.073  (0.073) | -0.026 (0.065) | 0.070  (0.063) | 0.024  (0.088) | -0.010  (0.094) | 0.028  (0.083) | 0.065  (0.090) |
| diff. South Kivu^b^ | 0.595*** (0.160) | 0.198  (0.171) | 0.373*** (0.133) | -0.062 (0.149) | 0.623*** (0.137) | 0.252* (0.145) | 0.196  (0.180) | 0.216  (0.179) | -0.003  (0.140) | 0.242  (0.169) |
| effect in South Kivu^c^ | 0.590*** (0.143) | 0.249  (0.156) | 0.388*** (0.113) | 0.011  (0.130) | 0.597*** (0.120) | 0.322** (0.131) | 0.221  (0.157) | 0.205  (0.152) | 0.026  (0.112) | 0.308**  (0.143) |
| Kivu baseline difference^d^ | 0.018  (0.073) | -0.066 (0.078) | -0.077 (0.061) | -0.184*** (0.060) | 0.211*** (0.063) | -0.018 (0.050) | -0.330***  (0.094) | -0.146  (0.096) | 0.407***  (0.066) | 0.119  (0.086) |
| controls | no | no | no | no | no | no | no | no | no | no |
| district FE | no | no | no | no | no | no | no | no | no | no |
| N | 629 | 648 | 629 | 648 | 629 | 648 | 647 | 658 | 658 | 658 |
| adj. R-sq | 0.152 | 0.076 | 0.080 | 0.033 | 0.175 | 0.154 | 0.092 | 0.033 | 0.307 | 0.000 |
| **diff-in-diff with controls** | | | | | | | | | | |
| effect in Burundi^e^ | -0.001 (0.069) | 0.058  (0.065) | 0.019  (0.067) | 0.078  (0.069) | -0.029 (0.059) | 0.070  (0.059) | 0.025  (0.085) | -0.009 (0.096) | 0.033  (0.081) | 0.065  (0.088) |
| diff. SouthKiv^f^ | 0.590*** (0.157) | 0.192  (0.168) | 0.369*** (0.133) | -0.067 (0.145) | 0.626*** (0.133) | 0.253* (0.141) | 0.210  (0.183) | 0.214  (0.180) | -0.007 (0.137) | 0.242  (0.162) |
| effect in SouthKiv^g^ | 0.590*** (0.141) | 0.249  (0.154) | 0.388*** (0.115) | 0.011  (0.127) | 0.597*** (0.120) | 0.322** (0.128) | 0.235  (0.162) | 0.205  (0.153) | 0.026  (0.110) | 0.308** (0.136) |
| Kivu baseline difference^h^ | 0.073  (0.125) | -0.162 (0.137) | -0.073  (0.111) | -0.336*** (0.110) | 0.221** (0.092) | -0.113 (0.093) | -0.488*** (0.135) | -0.138 (0.167) | 0.298*** (0.108) | 0.002  (0.150) |
| Controls | no | no | no | no | no | no | no | no | no | no |
| district FE | no | no | no | no | no | no | no | no | no | no |
| N | 629 | 648 | 629 | 648 | 629 | 648 | 647 | 658 | 658 | 658 |
| adj. R-sq | 0.229 | 0.163 | 0.158 | 0.131 | 0.284 | 0.256 | 0.170 | 0.025 | 0.349 | 0.067 |

Note: Standard errors in parentheses | see Tables 2 and A1 for the indicators that make indexs, and Table A2 for robustness checks | a, b, c, and d, are β_1_, β_2_, β_1_ + β_2_, and β_0_ in model 3. e-h, the same for model 4 (with γ instead of β) | ±. estimation for weighted and stratified sample, no adjusted R^2^.
