# Supplementary material for: Strengthening the community governance of healthcare services in ‘fragile’ settings: Evidence from Burundi and South Kivu, DR Congo
Source: PLOS Glob Public Health. 2023 Aug 15;3(8):e0001697. doi: 10.1371/journal.pgph.0001697 (PMC10427014; doi:10.1371/journal.pgph.0001697)
Supplement: S6 Table — (DOCX) [file pgph.0001697.s006.docx]

**S6 Table** Intent-to-Treat – indicators of HF management (robustness checks)

|  | (1) | (2) | (3) | (4) |
| --- | --- | --- | --- | --- |
| **Diff-in-diff without controls** | |  |  |  |
|  | stock-outs  of drugs | human  resources | infrastructure | finance |
| effect in Burundi^a^ | 0.051  (0.099) | 0.097  (0.120) | 0.076  (0.116) | -0.000  (0.075) |
| diff. South Kivu^b^ | 0.093  (0.301) | 0.183  (0.208) | 0.312  (0.257) | 0.014  (0.201) |
| effect in South Kivu^c^ | 0.143  (0.284) | 0.280*  (0.170) | 0.388*  (0.229) | 0.013  (0.186) |
| Kivu baseline difference^d^ | -0.730*** (0.132) | -0.239** (0.117) | -0.790*** (0.136) | 0.158  (0.097) |
| controls | no | no | no | no |
| district FE | no | no | no | no |
| N | 658 | 658 | 658 | 658 |
| adj. R-sq | 0.266 | 0.025 | 0.159 | 0.198 |
| **Diff-in-diff with controls** | |  |  |  |
| effect in Burundi^a^ | 0.050  (0.083) | 0.108  (0.098) | 0.074  (0.116) | 0.013  (0.065) |
| diff. South Kivu^b^ | 0.093  (0.255) | 0.171  (0.189) | 0.314  (0.247) | 0.280  (0.206) |
| effect in South Kivu^c^ | 0.143  (0.242) | 0.280* (0.162) | 0.388* (0.218) | 0.293  (0.196) |
| Kivu baseline difference^d^ | -1.525*** (0.185) | -0.270 (0.200) | -0.970*** (0.213) | -0.103 (0.158) |
| controls | yes | yes | yes | yes |
| district FE | yes | yes | yes | yes |
| N | 656 | 656 | 656 | 656 |
| adj. R-sq | 0.476 | 0.308 | 0.201 | 0.167 |
| **ANCOVA** |  |  |  |  |
| effect in Burundi^a^ | 0.062  (0.075) | 0.06  (0.077) | 0.078  (0.070) | 0.010  (0.053) |
| difference in South Kivu^b^ | -0.059 (0.163) | 0.153  (0.145) | 0.190  (0.159) | 0.124  (0.133) |
| Total effect in South Kivu^c^ | 0.002  (0.145) | 0.213* (0.123) | 0.268* (0.143) | 0.134  (0.122) |
| difference at baseline  (Kivu)^d^ | -0.373*** (0.129) | 0.055  (0.092) | -0.088 (0.118) | 0.247** (0.096) |
| controls | no | no | no | no |
| district FE | no | no | no | no |
| N | 329 | 329 | 329 | 329 |
| adj. R-sq | 0.440 | 0.372 | 0.116 | 0.217 |
| effect in Burundi^a^ | 0.062  (0.075) | 0.06  (0.077) | 0.078  (0.070) | 0.010  (0.053) |
| difference in South Kivu^b^ | -0.059 (0.163) | 0.153  (0.145) | 0.190  (0.159) | 0.124  (0.133) |

Note: Standard errors in parentheses | see Tables 2 and A1 for the indicators that make indexs, and Table A2 for robustness checks | a, b, c, and d, are β_1_, β_2_, β_1_ + β_2_, and β_0_ in model 3 | ±. estimation for weighted and stratified sample, no adjusted R^2^.
