# Supplementary material for: Strengthening the community governance of healthcare services in ‘fragile’ settings: Evidence from Burundi and South Kivu, DR Congo
Source: PLOS Glob Public Health. 2023 Aug 15;3(8):e0001697. doi: 10.1371/journal.pgph.0001697 (PMC10427014; doi:10.1371/journal.pgph.0001697)
Supplement: S7 Table — (DOCX) [file pgph.0001697.s007.docx]

**S7 Table** Heterogeneous effects: HFC composition (ANCOVA)

|  | (1) | (2) | (3) | (4) |
| --- | --- | --- | --- | --- |
|  | HFC rights  H = Kivu | HFC rights  H = ratio women  in HFC | HFC rights  H = ratio HFC members  with sec. education | HFC rights  H = ration HFC  members not farmers |
| Intervention^a^ | 0.140* (0.074) | 0.177  (0.155) | 0.058  (0.119) | 0.336***  (0.123) |
| H (source of  heterogeneity)^b^ | 0.119  (0.104) | -0.078  (0.326) | 0.374*  (0.192) | 0.339  (0.344) |
| Intervention*H^d^ | 0.659*** (0.147) | 0.200  (0.411) | 0.562*  (0.298) | -0.487  (0.594) |
| controls | No | No | No | No |
| district FE | No | No | No | No |
| N | 329 | 329 | 329 | 329 |
| adj. R-sq | 0.189 | 0.041 | 0.115 | 0.043 |

Note: standard errors in parentheses. Naïve p-values are reported: level of significance: <0.1, *<0.05, ***<0.001. | a, b, and d, are respectively, γ_1_, γ_2_, and γ_0_ in model 4.
