# Supplementary material for: Strengthening the community governance of healthcare services in ‘fragile’ settings: Evidence from Burundi and South Kivu, DR Congo
Source: PLOS Glob Public Health. 2023 Aug 15;3(8):e0001697. doi: 10.1371/journal.pgph.0001697 (PMC10427014; doi:10.1371/journal.pgph.0001697)
Supplement: S8 Table — (DOCX) [file pgph.0001697.s008.docx]

**S8 Table** Heterogeneous effects: management structure (ANCOVA)

|  | (1) | (2) | (3) | (4) |
| --- | --- | --- | --- | --- |
|  | HFC rights  H = Kivu | HFC rights  H = Faith-based HF | HFC rights  H = Cath. HF | HFC rights  H = Protestant HF |
| Intervention^a^ | 0.140*  (0.074) | 0.200***  (0.074) | 0.277***  (0.070) | 0.181**  (0.072) |
| H (source of  heterogeneity)^b^ | 0.119  (0.104) | -0.323***  (0.111) | -0.085  (0.172) | -0.494***  (0.083) |
| Intervention*H^d^ | 0.659***  (0.147) | 0.206  (0.164) | -0.206  (0.234) | 0.606***  (0.174) |
| controls | No | No | No | No |
| district FE | No | No | No | No |
| N | 329 | 329 | 329 | 329 |
| adj. R-sq | 0.189 | 0.062 | 0.055 | 0.066 |

Note: standard errors in parentheses. Naïve p-values are reported: level of significance: <0.1, *<0.05, ***<0.001. | a, b, and d, are respectively, γ_1_, γ_2_, and γ_0_ in model 4.
