# Supplementary material for: Strengthening the community governance of healthcare services in ‘fragile’ settings: Evidence from Burundi and South Kivu, DR Congo
Source: PLOS Glob Public Health. 2023 Aug 15;3(8):e0001697. doi: 10.1371/journal.pgph.0001697 (PMC10427014; doi:10.1371/journal.pgph.0001697)
Supplement: S9 Table — (DOCX) [file pgph.0001697.s009.docx]

**S9 Table** Heterogeneous effects: relationship to HF (ANCOVA)

|  | (1) | (2) | (3) |
| --- | --- | --- | --- |
|  | HFC rights  H = Kivu | HFC rights  H = HF-weighted ratio of households ‘facing staff’ | HFC rights  H = district-weighted ratio of households ‘facing staff’ |
| Intervention^a^ | 0.140*  (0.074) | 0.084  (0.079) | 0.015  (0.086) |
| H (source of  heterogeneity)^b^ | 0.119  (0.104) | -0.194  (0.293) | 0.224  (0.410) |
| Intervention*H^d^ | 0.659*** (0.147) | 1.854***  (0.490) | 2.727***  (0.622) |
| controls | No | No | No |
| district FE | No | No | No |
| N | 329 | 329 | 329 |
| adj. R-sq | 0.189 | 0.107 | 0.156 |

Note: standard errors in parentheses. Naïve p-values are reported: level of significance: <0.1, *<0.05, ***<0.001. | a, b, and d, are respectively, γ_1_, γ_2_, and γ_0_ in model 4.
