# Supplementary material for: Strengthening the community governance of healthcare services in ‘fragile’ settings: Evidence from Burundi and South Kivu, DR Congo
Source: PLOS Glob Public Health. 2023 Aug 15;3(8):e0001697. doi: 10.1371/journal.pgph.0001697 (PMC10427014; doi:10.1371/journal.pgph.0001697)
Supplement: S10 Table — (DOCX) [file pgph.0001697.s010.docx]

**S10 Table** Heterogeneous effects – multiple sources (ANCOVA)

|  | (1) | (2) | (3) | (4) | (5) | (6) |
| --- | --- | --- | --- | --- | --- | --- |
|  | HFC rights | HFC rights | HFC rights | HFC rights | HFC rights | HFC rights |
| Intervention^a^ | 0.140* (0.074) | 0.097 (0.117) | 0.116 (0.075) | 0.078 (0.103) | -0.001 (0.130) | 0.062 (0.103) |
| Intervention*  Kivu^b^ | 0.659*** (0.147) | 0.624*** (0.156) | 0.542*** (0.148) | 0.395 (0.424) | 0.271 (0.420) | 0.311 (0.421) |
| Intervention*  HFC schooling^a^ |  | 0.135 (0.301) |  |  | 0.222 (0.299) |  |
| Intervention*  Protestant HF^b^ |  |  | 0.541*** (0.147) |  | 0.559*** (0.156) | 0.535*** (0.147) |
| Intervention*  Face staff^c^ |  |  |  | 1.220 (1.763) | 0.976 (1.779) | 1.075 (1.762) |
| controls | No | No | No | No | No | No |
| district FE | No | No | No | No | No | No |
| N | 329 | 329 | 329 | 329 | 329 | 329 |
| adj. R-sq | 0.189 | 0.202 | 0.216 | 0.190 | 0.229 | 0.217 |

Note: standard errors in parentheses. Naïve p-values are reported: level of significance: <0.1, *<0.05, ***<0.001. | the model is model 4 with additional interaction terms | a. HFC schooling is ratio of HFC members with secondary education (see Table A6) | b. ‘Protestant HF’ is a binary variable taking the value 1 if the HF is a publicly-funded Protestant faith-based HF (see Table 7) | c. ‘face staff’ is the district-weighted proportion of surveyed households who said they would face the HF staff if they have a problem at their HF (see Tables 8 and A7)
