# Supplementary material for: Strengthening the community governance of healthcare services in ‘fragile’ settings: Evidence from Burundi and South Kivu, DR Congo
Source: PLOS Glob Public Health. 2023 Aug 15;3(8):e0001697. doi: 10.1371/journal.pgph.0001697 (PMC10427014; doi:10.1371/journal.pgph.0001697)
Supplement: S1 Text — (DOCX) [file pgph.0001697.s011.docx]

**S1 Text**. Evaluation framework

The main effects of the intervention are evaluated using a difference-in-difference model; this is to ensure that both HF and household-level are evaluated with the same approach. The basic specification is the following standard model:

$Y_{i,t}= c+t+I_{1}+\beta_{1}I_{1}t+\varepsilon_{i,t}$ (1)

where I is a binary variable denoting the intervention group. β_1_, the interaction between this variable and t, the binary variable identifying pre- and post-intervention periods, gives the effect. Robustness checks include adding district-level fixed effects (D_i_) and cX_i_, a set of key pre-intervention level covariates including the number of HFC member, the ratio of women, ratio of members who had attended secondary school, number of hills or villages represented, whether the HF is faith-based, the number of qualified nurses, water and electricity provisions, and the population of the HF catchment area. For models using household-level data, we also checked that the findings are robust to adding a vector cU_h,t_ containing person-level sociodemographic information in four items: age, gender, education and wealth (through a proxy: whether the interviewee lives off subsistence farming). Household-level data is weighted based on population-level characteristics (gender and age) reported in the latest Burundi and South Kivu census and taking account cluster sampling and stratification. A better specification when panel data is available, as is the case for our HF-level data but not for our household-level data, is an ANCOVA specification in which the outcome of interest is regressed on its lagged value [1]:

$Y_{i,1}= c+Y_{i,0}+\gamma_{1}I_{i}+\varepsilon_{i}$ (2)

where γ_1_ is the impact of the intervention. In this paper, we provide the difference-in-difference specification in the main text and the ANCOVA in the appendix when presenting side-by-side effects on both HF- and household-level outcomes. We present the ANCOVA results in the main text when only considering HF-level results (the difference-in-difference remain available in the appendix).

A key interest of this paper is that the RCT covers two countries. Estimating the effects for the intervention group each country is rather straightforward: a binary variable K taking the value 1 if the HF is located in South Kivu is introduced in either model. Model 1 becomes:

$Y_{i,t}= c+t+K_{i}+I_{1}+\beta_{0}K_{i}I_{1}+\beta_{1}I_{1}t+\beta_{2}I_{1}K_{i}t+\varepsilon_{i,t}$ (3)

where the effect in Burundi is given by β_1_ while South Kivu’s is β_1_ + β_2_. Model 2 becomes:

$Y_{i,1}= c+Y_{i,0}+{\gamma_{0}K}_{i}+\gamma_{1}I_{i}+\gamma_{2}K_{i}I_{i}+ \varepsilon_{i}$ (4)

where the effect in Burundi is γ_1_ while the effect in South Kivu is γ_1_ + γ_2_. All standard errors are adjusted for multiple hypotheses testing (family-wise error rate, FWER).

*References*

1. McKenzie D. Beyond baseline and follow-up: The case for more T in experiments. J Dev Econ. 2012;99: 210–221. doi:10.1016/j.jdeveco.2012.01.002
