# Supplementary material for: Strengthening the community governance of healthcare services in ‘fragile’ settings: Evidence from Burundi and South Kivu, DR Congo
Source: PLOS Glob Public Health. 2023 Aug 15;3(8):e0001697. doi: 10.1371/journal.pgph.0001697 (PMC10427014; doi:10.1371/journal.pgph.0001697)
Supplement: Attachment — Submitted filename: rebuttal.docx [file pgph.0001697.s012.docx]

Dear reviewers,

Thank you very much for your kind feedback on our submission. We note that reviewer 1 was very pleased with the paper and only recommended checking typos, which we did. Reviewer 2 recommended relatively limited changes, mostly in relation with the presentation of the methods and findings. These suggestions are very helpful for us to improve the paper. The table below provides a point-by-point response to the comments, but we are happy to confirm that we took all suggestions on board. We are aware that the previous version of the paper was possibly too technical in some respects and pushed most of the technical elements to the appendix. We improved the accessibility of the paper by replacing some of the most complicated tables with charts.

The most difficult point was the suggestion to separate out findings and discussion (reviewer 2 suggested either renaming sections or creating two different sections). We hope that we have found a solution that both clarifies the structure of the paper and, at the same time, keeps the flow (reviewer 1 stressed that flow was a strength of our paper). The main problem is that we have different stages in our argument/research. We tried presenting the findings of all stages –the preliminary results, the main effects that stress the difference between the two countries, and the exploration of the reasons that may explain such difference after the additional results– in one section and then move to a general discussion but such structure makes the paper more, not less, complicated to follow. Our approach was, therefore, to follow an approach similar to papers that include multiple studies: present the findings and the discussion separately, but stage by stage. We now have:

- A first sub-section presenting the situation (results) at baseline. There is no discussion here as they just confirm what was introduced in the background section.
- A second sub-section presenting the main results.
- A third sub-section discussing the main findings.
- A fourth section presenting the additional results. It is a mixed methods section and the discussion elements are very limited there.

The beginning of section 5 explains that structure to the reader.

We remain open to try other structures. Please also note that our paper is mixed methods, cross country, and with an empirical methodology guided some of the analysis –all of this really complicates attempts to have it fit a more standard structure.

We hope that you will like the new version of our paper and thank you again for your suggestions. We remain open to continuing the discussion.

Sincerely Yours,

| *Reviewer 1* | Thank you for your very kind comments. The paper took a long time to become mature and we are very pleased that you liked it. |
| --- | --- |
| *Reviewer 2* |  |
| *In this version, the reader can find results in the introduction as well as discussion elements in the results. Please make sure to move all results to the corresponding section (examples include lines 61 – 65 “In Burundi, only the organization of the HFC … quality of services” and table 1) for ease of reading and understanding among readers.* | Thank you. We moved the section and table you mentioned to the first part of our results. |
| *Some statements related to the context characteristics (e.g. first paragraph of section 3) are presented with any references. Please add relevant citations.* | Sorry for missing those references. Section 3 is mostly describing the legal and administrative context and we added references to the relevant MoH guidelines and documents. |
| *The presentation of methods requires some clarifications/modifications. The main concern is the lack of clarity about the research design of the quantitative component. Authors refers to it as an RCT (randomized clinical? trial) but they do not present all the details: how was the randomization process conducted/secured? What is the ratio given that 168 vs 83 facilities were selected (vs not) in Burundi? Authors are invited to explain this more or to identify this component as non-randomized trial if applicable.* | This is indeed a Randomised Control Trial (RCT), and we provided clearer explanations in the paper. The text now clarifies that the selection of the health facilities was random among all HFs in the zones covered by the study (those not selected were the control group). Endnote ii provides the technical details. The ratio is not exactly half in each group, but this has no bearing on the validity of the experiment (again, footnote ii goes into the details and explains why we decided not to go for 50/50 in the case of Burundi –these details are informative, but we don’t think they belong to the main text). |
| *The evaluation framework can be an appendix unless presented in simplified way to readers with no strong statistics background.*  *The actual data collection methods especially surveys need to be clarified (with tools or variables can be added in the appendices) as the paragraph under table 2 is not clear. For instance, which survey informed table 3 results? What is the sample size of this (or these) surveys? How was data analysed?*  *Also, does table 3 shows no statistical difference between the two groups for all variables as the footnotes show an explanation of level of significance for (*), (**?) and (***) but without using these signs in the table. Could you please mention it explicitly if none of the comparison had a p<0.1)* | Thank you, we simplified this section and moved the bulk of it to the appendix. We also decided to present most results in graphical form to make it simpler for readers with less strong statistical background. Of course, all the details remain available in the appendix.  We are, of course, committed to releasing the data and instruments and indicated so in our original submission (we understand this is also a requirement of the journal) –our understanding is that this is normally separate from the appendix. Table A2 (formerly Table 2) provides indications relative to sample sizes. Sources have been added to chart and table notes. The evaluation framework provides details on the data analysis for most tables (and we systematically report what the coefficients correspond to). We also amended the data source and outcomes sections to clarify the sources used for each dimension we assessed. This information is also provided in detail in table A1.  We amended Table 3 to state that none of the comparison had a p<0.1. We also pushed Table 3 to the appendix to simplify the paper. |
| *On the main outcomes, there is a confusion whether the study included 2 or 3 outcomes under accountability, as table 2 mentions 3 but the subsequent tables mention HFC org. and HFC reach.*  *Overall, the tables are difficult to read.* | Thank you, there are two outcomes under accountability, we clarified that point.  See our earlier response. We replaced most tables with charts. |
| *The results section includes discussion of many findings with reference to other studies in the literature. Authors may want to consider writing a discussion section where these points are noted or (in case of a reason for not to) to call their results section as “results and discussion”.* | Thank you, see our general comment above. |
